# Ergolide mediates anti-cancer effects on metastatic uveal melanoma cells and modulates their cellular and extracellular vesicle proteomes

**DOI:** 10.12688/openreseurope.15973.1

**Published:** 2023-06-08

**Authors:** Husvinee Sundaramurthi, Valentina Tonelotto, Kieran Wynne, Fiona O'Connell, Eve O’Reilly, Marcel Costa-Garcia, Csenger Kovácsházi, Agnes Kittel, Simone Marcone, Alfonso Blanco, Eva Pallinger, Szabolcs Hambalkó, Jose Maria Piulats Rodriguez, Péter Ferdinandy, Jacintha O'Sullivan, David Matallanas, Lasse D. Jensen, Zoltán Giricz, Breandán N. Kennedy

**Affiliations:** 1 UCD Conway Institute, University College Dublin, Dublin, Leinster, Ireland; 2 UCD School of Biomolecular and Biomedical Science, University College Dublin, Dublin, Leinster, Ireland; 3 Systems Biology Ireland, University College Dublin, Dublin, Leinster, Ireland; 4 UCD School of Medicine, University College Dublin, Dublin, Leinster, Ireland; 5 Xenopat S.L., Business Bioincubator, Bellvitge Health Science Campus, Barcelona, 08907 L'Hospitalet de Llobregat, Spain; 6 Department of Surgery, Trinity Translational Medicine Institute, Trinity St. James's Cancer Institute, St. James's Hospital, Trinity College Dublin, Dublin, Ireland; 7 Medical Oncology Department, Catalan Institute of Cancer (ICO), IDIBELL-OncoBell, Barcelona, Spain; 8 Department of Pharmacology and Pharmacotherapy, Semmelweis University, Budapest, Hungary; 9 Pharmahungary Group, Szeged, Hungary; 10 Institute of Experimental Medicine, Budapest, Hungary; 11 Department of Genetics and Immunobiology, Semmelweis University, Budapest, Hungary; 12 BioReperia AB, Linköping, Sweden

**Keywords:** Metastatic uveal melanoma, ergolide, extracellular vesicles, BRCA2 and CDKN1A Interacting Protein, Chitinase Domain Containing 1

## Abstract

**Background:** Uveal melanoma is a poor prognosis cancer. Ergolide, a sesquiterpene lactone isolated from *Inula Brittanica*, exerts anti-cancer properties. The objective of this study was to *1)* evaluate whether ergolide reduced metastatic uveal melanoma (MUM) cell survival/viability *in vitro* and *in vivo*; and *2)* to understand the molecular mechanism of ergolide action.

**Methods:** Ergolide bioactivity was screened via long-term proliferation assay in UM/MUM cells and in zebrafish MUM xenograft models. Mass spectrometry profiled proteins modulated by ergolide within whole cell or extracellular vesicle (EVs) lysates of the OMM2.5 MUM cell line. Protein expression was analyzed by immunoblots and correlation analyses to UM patient survival used The Cancer Genome Atlas (TCGA) data.

**Results:** Ergolide treatment resulted in significant, dose-dependent reductions (48.5 to 99.9%; *p*<0.0001) in OMM2.5 cell survival *in vitro* and of normalized primary zebrafish xenograft fluorescence (56%; *p*<0.0001) *in vivo*, compared to vehicle controls. Proteome-profiling of ergolide-treated OMM2.5 cells, identified 5023 proteins, with 52 and 55 proteins significantly altered at 4 and 24 hours, respectively ( *p*<0.05; fold-change >1.2). Immunoblotting of heme oxygenase 1 (HMOX1) and growth/differentiation factor 15 (GDF15) corroborated the proteomic data. Additional proteomics of EVs isolated from OMM2.5 cells treated with ergolide, detected 2931 proteins. There was a large overlap with EV proteins annotated within the Vesiclepedia compendium. Within the differentially expressed proteins, the proteasomal pathway was primarily altered. Interestingly, BRCA2 and CDKN1A Interacting Protein (BCCIP) and Chitinase Domain Containing 1 (CHID1), were the only proteins significantly differentially expressed by ergolide in both the OMM2.5 cellular and EV isolates and they displayed inverse differential expression in the cells versus the EVs.

**Conclusions:** Ergolide is a novel, promising anti-proliferative agent for UM/MUM. Proteomic profiling of OMM2.5 cellular/EV lysates identified candidate pathways elucidating the action of ergolide and putative biomarkers of UM, that require further examination.

## Plain language summary

The most common form of adult eye cancer is uveal melanoma (UM). Once UM cancer cells spread to organs in the rest of the body, metastatic UM (MUM), there is a poor prognosis for patients with only one approved drug treatment. Hence, it is vital to better understand the cellular and extracellular proteins that regulate UM pathology in order to uncover biomarkers of disease and therapeutic targets. In this original study, we demonstrate a compound called ergolide is capable of severely reducing the metabolic activity and growth of UM cancer cells, grown as isolated monolayers. Ergolide was also able to reduce the growth of human MUM cells growing as tumors in transplanted zebrafish larvae. We identify that ergolide alters specific proteins found in the human UM cells. These proteins once analyzed in detail offer opportunities to understand how new treatment strategies can be developed for UM.

## Introduction

Uveal melanoma (UM) is the most common adult, intraocular cancer ^1, 2^. Primarily originating in the uveal tract (choroid, ciliary body and iris), UM presents with a poor survival prognosis and a high mortality rate of 92% within two years, once the disease metastasizes ^3^. Unfortunately, there are limited therapeutic options available for metastatic UM (MUM). Treatments under investigation for MUM have included site-directed therapies, conventional chemotherapeutic drugs, immune checkpoint inhibitors, targeted therapy and/or surgical resection, and although promising, limited long-term benefits were observed in the majority of patients ^4– 7^. Tebentafusp (an immunotherapy drug), received FDA and EMA approval in 2022 for the treatment of a sub-cohort (HLA-A*0201 positive) of UM patients ^8^. However, there is still an unmet need to identify novel biomarkers for early MUM diagnosis and to discover novel therapeutic targets and drugs for treatment of MUM. Currently, there are multiple clinical trials in various phases ongoing evaluating single agents or combinatorial therapies, immunotherapies for MUM ^7, 9, 10^.

Ergolide, a sesquiterpene lactone, isolated from dried flowers of a plant in the *Inula* genus, is a compound being analyzed for anti-cancer properties ^11, 12^. As traditional Chinese medicines, some *Inula* species are used for anti-inflammatory, antiemetic, diuretic and anti-bacterial indications ^12– 14^. A handful of pre-clinical studies report ergolide presenting with anti-cancer properties including cytotoxicity, leading to inhibition of cell proliferation and apoptosis ^15– 17^. Mechanistically, ergolide induced apoptosis in Jurkat cells by inhibiting the NF-κB signaling pathway, leading to the suppression of anti-apoptotic proteins ^16^. Moreover, ergolide is reported to inhibit NF-κB in RAW 264.7 macrophages and Hela cells ^18, 19^. Yami *et al.*, also demonstrated that ergolide reduced the viability of multiple leukemic cell lines through cell cycle arrest and induction of apoptosis ^17^. Collectively, these preliminary findings highlight the therapeutic potential of ergolide as an anti-cancer agent that warrants further evaluation. However, an *omics*-based approach has not been undertaken to investigate molecular pathways modulated by ergolide.

Given the promising results observed in cancer cell lines we hypothesized that ergolide may present with anti-proliferative effects in UM/MUM cells. In our study, we set out to evaluate the efficacy of ergolide as an anti-cancer drug in MUM cells. Results from our pre-clinical studies highlighted that ergolide treatment elicited anti-cancer benefits in MUM cells *in vitro*; and *in vivo* using zebrafish xenograft models. Proteome profiling of ergolide treated MUM cells helped understand the underlying molecular mechanism of ergolide in MUM cells. Furthermore, while extracellular vesicles (EV)-related processes are modulated in cancer, little is known about EVs in the context of UM/MUM disease pathogenesis, prognostication or therapeutic potential ^20– 22^. Therefore, we assessed changes to the proteome profile of EVs in MUM cells and observed that proteins related to the proteasomal pathway were significantly downregulated in EVs isolated from ergolide treated OMM2.5 cells. Our data highlights the prospective of ergolide as a potential therapeutic drug against MUM cell survival that needs further assessment. 

## Methods

### Cell culture

Mel285, Mel270 and OMM2.5 cells (kindly provided by Dr. Martine Jager, Leiden, The Netherlands) were cultured and passaged (no more than 20 passages) in complete media containing RPMI 1640 with GlutaMAX™ Supplement (Gibco; Waltham, MA, USA) + 10% fetal bovine serum (FBS; Gibco) + 2% penicillin–streptomycin (PEST) and incubated at 37°C with 5% CO _2_, unless otherwise stated ^23– 26^.

### MTT assay

Cell metabolism, an indirect measure of viability, was determined using MTT (3-(4,5-dimethylthiazol-2-yl)-2,5-diphenyltetrazolium bromide (#M5655), Sigma-Aldrich; St. Louis, MO, USA) assay. Briefly, 5 × 10 ^3^ cells/well were seeded into 96-well plates and allowed to adhere overnight. Cells were treated in triplicates with either 0.5% DMSO (vehicle control), 15% H _2_O _2_ (positive control) or 0.5–10 µM ergolide (#HY-N6893; MedChemExpress (MCE); NJ, USA), prepared in complete media for 96 hours. Once drug solution was removed, MTT dye and serum-free media were added in a 1:10 ratio, to each well and incubated in the dark for 2.5 h at 37°C. Then, 100% DMSO (1:1 ratio) was added to each well and absorbance measured at 570 nm using a SpectraMax® M2 microplate reader (Molecular Devices Corporation, Sunnyvale, CA, USA). One-way ANOVA with Dunnett's Test for Multiple Comparisons statistical analysis and the IC _50_ for ergolide was calculated using Graph Pad Prism v8.00 for Windows (GraphPad Software, San Diego, CA, USA).

### Clonogenic assay

Ergolide clonogenic experiments were performed using a previously established methodology and the same DMSO vehicle controls were used for quantification of colonies ^26^. Briefly, cells were seeded into 6-well plates at 1.5 × 10 ^3^ cells/mL (final volume 2 mL). The cells were treated in duplicate with either 0.5% DMSO or ergolide at 1–10 μM, in complete media and incubated at 37°C with 5% CO _2 _for 96 h. Cells were washed twice with 1 X phosphate-buffered saline (PBS; Lonza; Basel, Switzerland) and fresh complete media was added to plates and incubated for 10 days at 37°C with 5% CO _2_. Plates were washed with PBS and cells fixed with 4% paraformaldehyde and stained with 0.5% crystal violet solution (Pro-Lab diagnostics PL700; Richmond Hill, ON, Canada). Plates were imaged using GelCount™system (Oxford Optronix; Oxford, UK) and analyzed with ColonyCountJ Plugin (kindly shared by Dr. Dharmendra Kumar Maurya, Mumbai, India) in ImageJ v1.53e ^27^. Experiments were performed in triplicates. One-way ANOVA with Dunnett’s Test for Multiple Comparisons analysis was performed in GraphPad Prism v7.00 for Windows (GraphPad Software, San Diego, CA, USA).

### Drug treatment of OMM2.5 zebrafish xenograft models

The zebrafish xenograft methodology is as reported previously ^26^. Zebrafish rearing and husbandry were performed in accordance with ethical regulations of the Linköping Animal Research Ethics Committee. Only larval, and not animal forms, of zebrafish were used in the study. Experiments were performed concurrently with a previously published ACY-1215 study, vehicle controls used are the same samples for data analysis ^26^. An additional treatment arm of 2.5 μM ergolide was included in the study. Toxicity of ergolide was pre-tested in 2 day old zebrafish larvae, whereby a total of 8 larvae (4 larvae/well) were treated with either 0.5% DMSO or increasing concentrations of 0.5 – 10 μM ergolide for 3 days at 35°C, followed by imaging. Student’s T test statistical analyses were performed using GraphPad Prism v7.00.

### Proteomics sample preparation and Mass Spectrometry analysis

1 × 10 ^6^ OMM2.5 cells were seeded in triplicate wells and drug treated for 4 or 24 hours with 0.5% DMSO or 2.5 µM ergolide. Four independent experiments were performed, and protein isolated from cell lysates in accordance with PreOmics iST 8X for protein/proteomics preparation kit manufacturer’s instructions (#P.O.00001, PreOmics GmbH; Martinsried, Germany). The samples were analyzed by the UCD Conway Institute Mass Spectrometry Resource (MSR) on a Thermo Fisher Scientific Inc. Q Exactive mass spectrometer connected to a Dionex Ultimate 3000 (RSLCnano) chromatography system. Peptides were separated on C18 home-made column (C18-AQ Dr. Maisch Reprosil-Pur 100 × 0.075 mm × 3 μm) over 120 min at a flow rate of 250 nL/min with a linear gradient of increasing ACN from 1% to 27%. The mass spectrometer was operated in data dependent mode; a high resolution (70,000) MS scan (300–1600 m/z) was performed to select the twelve most intense ions and fragmented using high energy C-trap dissociation for MS/MS analysis.

Raw data from the Q Exactive was processed using MaxQuant (version 2.0.3.0) ^28, 29^ incorporating the Andromeda search engine ^30^. To identify peptides and proteins, MS/MS spectra were matched against Uniprot homo sapiens database (2021_03) containing 78,120 entries. All searches were performed using the default setting of MaxQuant, with trypsin as specified enzyme allowing two missed cleavages and a false discovery rate of 1% on the peptide and protein level. The database searches were performed with carbamidomethyl (C) as fixed modification and acetylation (protein N terminus) and oxidation (M) as variable modifications. For the generation of label free quantitative (LFQ) ion intensities for protein profiles, signals of corresponding peptides in different nano-HPLC MS/MS runs were matched by MaxQuant in a maximum time window of 1 min ^31^. Perseus software was used to process the data and create heatmaps ^32^. Student’s T Test was utilized for statistical analysis and multiple corrections was not performed in this study. ClueGo (v2.5.8) ^33^ and Cluepedia (v1.5.8) ^34^ plugins in Cytoscape (v3.8.2) ^35^ with the Homo sapiens (9606) marker set was utilized for GO:Biological processes pathway analysis of enriched proteins ^26^. Functional Enrichment analysis tool (FunRich v3.1.3) was used to create Venn diagrams using associated gene names identified ^36– 38^.

### ELISA inflammatory panel

Conditioned media from OMM2.5 cells treated for 4 and 24 hours with 0.5% DMSO or 2.5 µM ergolide was collected (triplicate) and stored at -80°C until use. Protein was isolated from the cell lysate and concentrations measured using BCA assay (Pierce; ThermoFisher Scientific; Waltham, MA, USA). Media was processed according to MSD (Meso Scale Discovery; Meso Scale Diagnostics, Rockville, MD, USA) multiplex protocol. To assess angiogenic, vascular injury, pro-inflammatory, cytokine and chemokine secretions from cell supernatants, a 54-plex ELISA kit separated across 7 plates were used (#K15248D-1, Meso Scale Diagnostics, USA). The multiplex kit was used to quantify the secretions of CRP, Eotaxin, Eotaxin-3, FGF(basic), Flt-1, GM-CSF, ICAM-1, IFN-γ, IL-10, IL-12/IL-23p40, IL-12p70, IL-13, IL-15, IL-16, IL-17A, IL-17A/F, IL-17B, IL-17C, IL-17D, IL-1RA, IL-1α, IL-1β, IL-2, IL-21, IL-22, IL-23, IL-27, IL-3, IL-31, IL-4, IL-5, IL-6, IL-7, IL-8, IL-8 (HA), IL-9, IP-10, MCP-1, MCP-4, MDC, MIP-1α, MIP-1β, MIP-3α, PlGF, SAA, TARC, Tie-2, TNF-α, TNF-β, TSLP, VCAM-1, VEGF-A, VEGF-C and VEGF-D. All assays were run as per the manufacturer’s recommendation, an overnight supernatant incubation protocol was used for all assays except Angiogenesis Panel 1 and Vascular Injury Panel 2 which were run according to the same-day protocol. Cell supernatants were run undiluted on all assays except Vascular Injury Panel 2, where a one in four dilution was used, as per previous optimization experiments. Secretion data for all factors were normalized to cell lysate protein content. Statistical analysis Student’s T Test was performed using GraphPad Prism v7.00.

### Extracellular vesicles sample preparation

OMM2.5 cells were cultured in complete media containing RPMI 1640 (Gibco) + 10% fetal bovine serum (Corning®; Corning, NY, USA) + 1 X Antibiotic-Antimycotic (Corning®) + 4 mM L-Glutamine (Corning®) and incubated at 37°C with 5% CO _2_. OMM2.5 cells were seeded into 8 – 12 T175 flasks at a seeding density of 4×10 ^6^ cells/flask in 25 ml of complete media per flask and incubated at 37°C with 5% CO _2_ for 24 h (N = 4). Then cells were washed with PBS and treated with either 0.1% DMSO or 2.5 µM ergolide in 20 ml serum-free media/flask. Conditioned media from each treatment group was collected after 24 h into 50 mL conical tubes by pooling media from four flasks. Medium was centrifuged at 300×g, 4°C for 10 min with swinging bucket rotor (Hettich, #1494). Supernatant was transferred to new tubes and re-centrifuged for 5 min at 2500×g, 4°C, then at 13,500×g, 4°C for 40 min. Finally, supernatant was ultracentrifuged for 3 h at 174,900×g, 4°C. Supernatant was discarded, and EV pellets resuspended in 120–145 μl of 0.2 μm filtered PBS. Isolated EV samples were stored at - 80°C after measurement of protein concentration (Nanodrop) and particle size distribution by nanoparticle tracking analysis (NTA). In parallel, drug treated cells were trypsinized, and collected. Cell pellet was resuspended in RIPA buffer (Cell Signaling Technologies; Danvers, MA, USA) and stored at - 20°C.

### Extracellular vesicles mass spectrometry and data analysis

30 μg of EV samples (N = 4 for each treatment condition) was prepared after quantification with micro BCA™ Protein Assay Kit (#23235, Thermo Fisher Scientific Inc.), volume adjusted with PBS and vacuum dried. Following trypsin digestion, the samples were cleaned using C18 HyperSep SpinTips (Thermo Fisher Scientific Inc.). Each sample was analysed in duplicate on a Bruker timsTof Pro mass spectrometer (Bruker Daltonics, Bremen) connected to a Bruker nanoElute nano-lc chromatography system (Bruker Daltonics). Tryptic peptides were resuspended in 0.1% formic acid and injected onto a pepmap100 C18 5 μm trap column (0.3mm × 5mm) (Thermo Fisher Scientific Inc.) prior to separation with an increasing acetonitrile gradient using an Aurora UHPLC column (25 cm × 75 μm ID, C18, 1.6 μm, (Ionopticks) ^35^. The mass spectrometer was operated in positive ion mode, with a capillary voltage of 1500 V, dry gas flow of 3 l/min and a dry temperature of 180°C. All data was acquired with the instrument operating in trapped ion mobility spectrometry (TIMS) mode. Trapped ions were selected for ms/ms using parallel accumulation serial fragmentation (PASEF). A scan range of 100–1700 m/z was performed at a rate of 5 PASEF MS/MS frames to 1 MS scan with a cycle time of 1.03 seconds ^39^.

The mass spectrometer raw data was searched against the *Homo Sapiens* subset of the Uniprot Swissprot database (reviewed) using the search engine Maxquant (release 2.0.2.0). In brief, specific parameters were used (Type: TIMS DDA, Fixed mods; Carbamidomethyl (C), Variable mods; Oxidation (M), Acetyl (Protein N-term). Each peptide used for protein identification met specific Maxquant parameters, *i.e.,* only peptide scores that corresponded to a false discovery rate (FDR) of 0.01 were accepted from the Maxquant database search. Ratio of LFQ average for each condition was calculated and fold change was used together with two-tailed, equal distribution Student’s T test employed to test for statistical difference. Proteins that showed a >1.5 fold ratio between conditions change and *p*< 0.05, were determined as enriched ^40^. Heatmaps were generated with Cluster 3.0 using Pearson correlation uncentered, with average linkage ^41^ and visualized with Java TreeView software ^42^. Enriched pathway analysis performed using AmiGO 2 v2.5.17, STRING v11.5; Cytoscape and Venn diagrams created with FunRich tool as above.

### NTA analysis

Nanoparticle tacking analysis (NTA) measurements were performed on Zeta View PMX 110 (Particle Metrix, Meerbusch, Germany). Samples were diluted in 0.2 μm filtered PBS to have 50–300 visible particles. Automated measurement was acquired with 11 positions throughout the measurement cell, with two cycles of readings at each position. Readings for which the software recommended exclusion were excluded from the final evaluation. Instrument parameters were set as: temperature of 25°C, sensitivity of 75, frame rate of 7.5 frames per second, shutter speed of 100. Post-acquisition parameters were set as: minimum brightness 20, minimum size 5 pixels, maximum size 1000 pixels. Results were multiplied by the dilution factor and particle concentration and medial particle size was calculated. To visualize data, it was smoothed with Loess regression and presented as mean ±SEM using R programming language.

### Transmission electron microscopy

Visualization of EVs from ergolide-treated cells was accomplished by transmission electron microscopy (TEM) of resin-embedded samples. EV samples were centrifuged at 120,000× *g*, 4°C, for 1 h in 5 mL polypropylene centrifugation tubes. Pellets were fixed with 4% paraformaldehyde, postfixed in 1% osmium tetroxide (OsO _4_) for 20 min. After rinsing with distilled water, they were dehydrated by ethanol including block staining with 1% uranyl-acetate in 50% ethanol for 30 min and embedded in Taab 812 (Taab). Ultrathin sections were analyzed with a Hitachi 7100 (Hitachi Ltd, Tokyo, Japan) electron microscope equipped with Veleta, a 4 megapixel side-mounted transmission EM CCD camera (Olympus, Tokyo, Japan).

### Western blot analysis

For EVs, samples were lysed in RIPA buffer (Cell Signaling Technologies) supplemented with 1 mM phenylmethylsulfonyl fluoride (PMSF) (Roche; Basel, Switzerland), 0.1 mM sodium fluoride, and complete protease inhibitor cocktail (Roche). Equal volumes from each sample were mixed with 1/4 volume of Laemmli buffer containing β-mercaptoethanol (Thermo Fisher Scientific Inc.), loaded on tris-glycine sodium dodecyl sulfate-polyacrylamide gels (Bio-Rad, Hercules, CA, USA), and electrophoresed. Proteins were transferred onto polyvinylidene fluoride (PVDF) membranes (Bio-Rad) which were blocked with 5% bovine serum albumin (BSA; Bio-Rad) in tris-buffered saline containing 0.05% Tween-20 (TBST) for 2 h at room temperature. Primary antibodies used were anti-TSG101 (#ab83, Abcam, Cambridge, UK), anti-Alix (#sc-53540, Santa Cruz Biotechnology, Dallas, Texas, USA), anti-CD81 (#sc-166029, Santa Cruz Biotechnology, Dallas, Texas, USA) and anti-HSP70 (#sc-66049, Santa Cruz Biotechnology, Dallas, Texas, USA). The presence of individual contaminating organelles was detected using the Organelle Detection Western Blot Cocktail (#ab133989, Abcam; Cambridge, UK) comprising of anti-sodium-potassium ATPase (plasma membrane), anti-ATP5A (mitochondria), anti-GAPDH (cytosol) and anti-Histone-H3 (nucleus) antibodies. Horseradish peroxidase-conjugated secondary antibodies (#7076, Cell Signaling Technology) were used to detect the proteins. Blots were visualized using enhanced chemiluminescence kit (#170-5061, Bio-Rad) by Chemidoc XRS ^+^ (Bio-Rad) and analyzed with Image LabTM software (Bio-Rad).

For cell lysates, protein concentration was quantified using Pierce BCA Protein Assay Kit (#23227, Thermo Fisher Scientific Inc.) and 10 μg of each sample was loaded and run on a Mini-PROTEAN TGX gel (Bio-Rad). Protein samples were transferred via wet transfer onto a PVDF membrane and blocked with 5% BSA in TBST for 1 h at room temperature. Membrane was incubated with either HMOX1 (#10701-1-AP; Proteintech), GDF15 (#27455-1-AP; Proteintech) or β-ACTIN (#sc-47778, Santa Cruz Biotechnology), overnight at 4°C. Membranes were washed thrice with TBST for 10 mins each and incubated with either anti-rabbit (#7074; Cell Signaling) or anti-mouse (#7076; Cell Signaling) secondary antibodies for 1 h at room temperature. Membranes were washed as stated previously and imaged using Pierce ECL Western Blotting Substrate (Thermo Fisher Scientific Inc.) on Vilber Fusion FX Spectra imager.

### TCGA data analysis

Clinical and mutational data of primary uveal tumors was collected from TCGA-UM dataset included in The Cancer Genome Atlas (TCGA; n = 80). Annotated mutational data was downloaded from the cBioPortal ^43^. RNA-seq data was downloaded in fragments per kilobase per million (FPKM), then converted to log2 scale. Expression between chromosome 3 monosomic or disomic patients was statistically compared using the Mann-Whitney U test. Survival analysis was performed using Overall Survival (OS) or Disease-Free Survival (DFS) from annotation. Kaplan–Meier curves were plotted to represent the result and log-rank test was computed.

##  Results

### Ergolide exerts anti-proliferative effects in MUM cells

**Figure 1.**
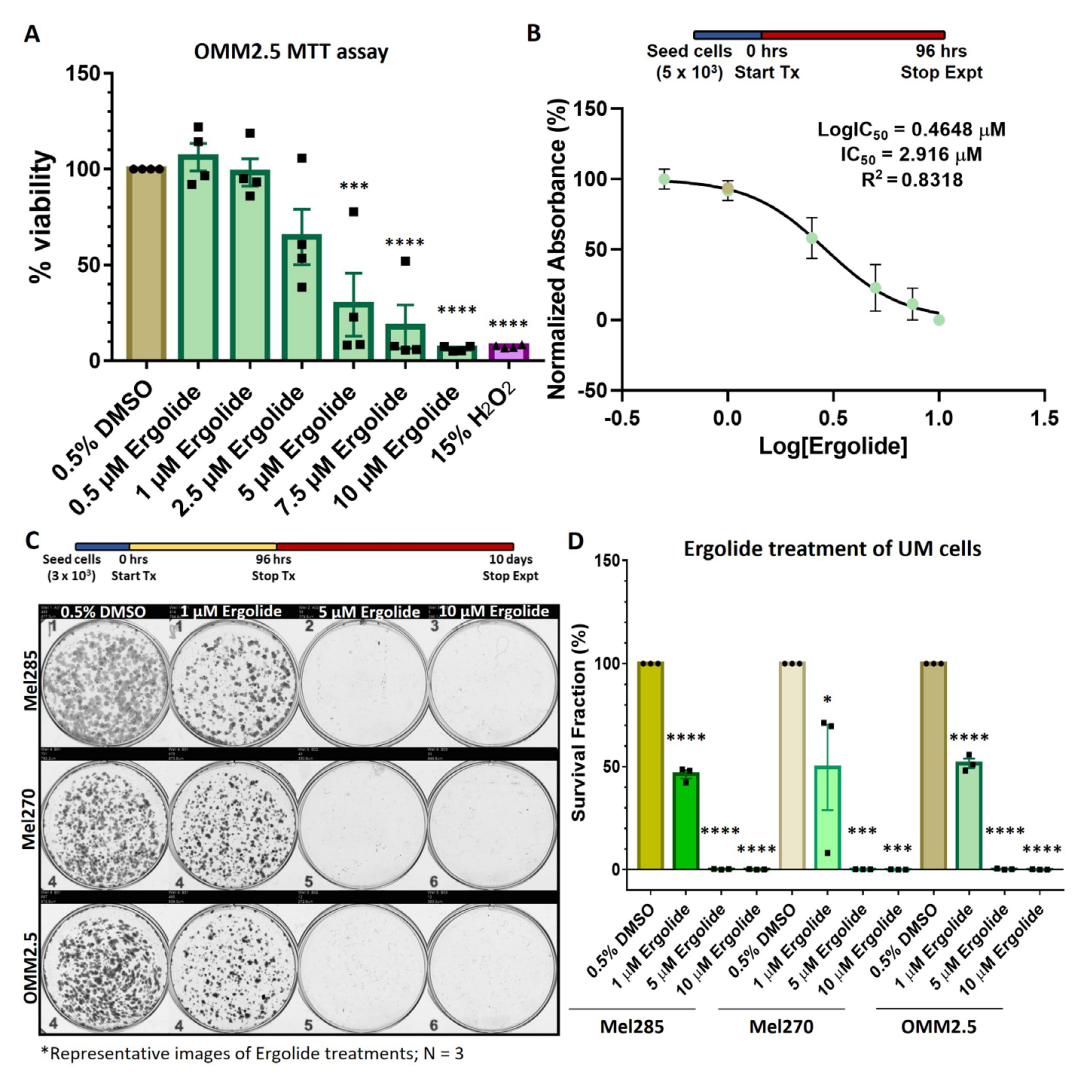
Ergolide treatment significantly reduced UM/MUM cell viability. ( **A**, **B**) Dose-response analysis and determination of IC _50_ of ergolide in OMM2.5 cells as 2.9 μM via the MTT assay. ( **C**, **D**) Long-term survival of Mel285, Mel270 and OMM2.5 clones were significantly (****, adjusted *p*=0.0001) reduced in ergolide treated samples compared to vehicle control (N = 3). Statistical analysis performed using One-way ANOVA with Dunnett's Test for Multiple Comparisons.

As more effective treatments are needed for UM, we investigated if the reported anti-cancer properties of ergolide are observed in UM cell lines. The effect of ergolide treatment on UM cell viability was analyzed in the OMM2.5, UM liver metastatic cell line. Cells were treated with either increasing concentrations (0.5 - 10 μM) of ergolide or 0.5% DMSO (vehicle control) or 15% hydrogen peroxide (H _2_O _2_, positive control) for 96 hours ( Figure 1). Significant dose-dependent reductions averaging 70.66%, 82.18% and 93.61% ( *p* = 0.0001) respectively, in cell metabolism were observed when treated with 5, 7.5 or 10 μM ergolide, in comparison to vehicle controls ( Figure 1A). H _2_O _2_ treatment of OMM2.5 cells resulted in an average of 92.43% reduction in viable cells compared to vehicle controls. For MTT assays, the calculated IC _50_ of ergolide in OMM2.5 cells was 2.9 μM ( Figure 1B).

Furthermore, the anti-proliferative effect of ergolide was determined in both primary (Mel285 and Mel270) and metastatic (OMM2.5) UM derived cell lines using clonogenic assays. Mel270 (choroidal melanoma) and OMM2.5 cell lines are derived from the same patient and harbor a GNAQ ^Q209P^ mutation, while Mel285 (choroidal melanoma) has an unknown mutation ^24^. All three cell lines were treated with 1, 5 or 10 μM ergolide (encompassing the MTT IC _50_) for 96 hours and subsequently cultured for 10 days prior to analysis. A significant dose-dependent reduction in cell proliferation was noted across the three cell lines at all concentrations tested ( Figure 1C and 1D). 1, 5, or 10 μM ergolide treatment resulted in an average reduction of surviving colonies by 53.71% ( *p*=0.0001), 99.8% ( *p*=0.0001), 99.9% ( *p*=0.0001), respectively in Mel285 cells; and average reductions of 50.39% ( *p*=0.0224), 99.85% ( *p*=0.0004) and 99.96% ( *p*=0.0004), respectively, in Mel270 cells compared to vehicle controls ( Figure 1D). In OMM2.5 cells, a significant average reduction of 48.45% ( *p*=0.0001), 99.73% ( *p*=0.0001) and 99.93% ( *p*=0.0001) in surviving colonies was observed following 1, 5, or 10 μM ergolide treatment in comparison to vehicle controls ( Figure 1D). These findings confirm that ergolide attenuates long-term proliferation of primary and metastatic UM cell lines. As our focus was to identify novel therapeutic options for metastatic UM, all follow-up experiments were performed in OMM2.5 cells. 

### Ergolide produces anti-cancer effects *in vivo* in OMM2.5 zebrafish xenografts

**Figure 2.**
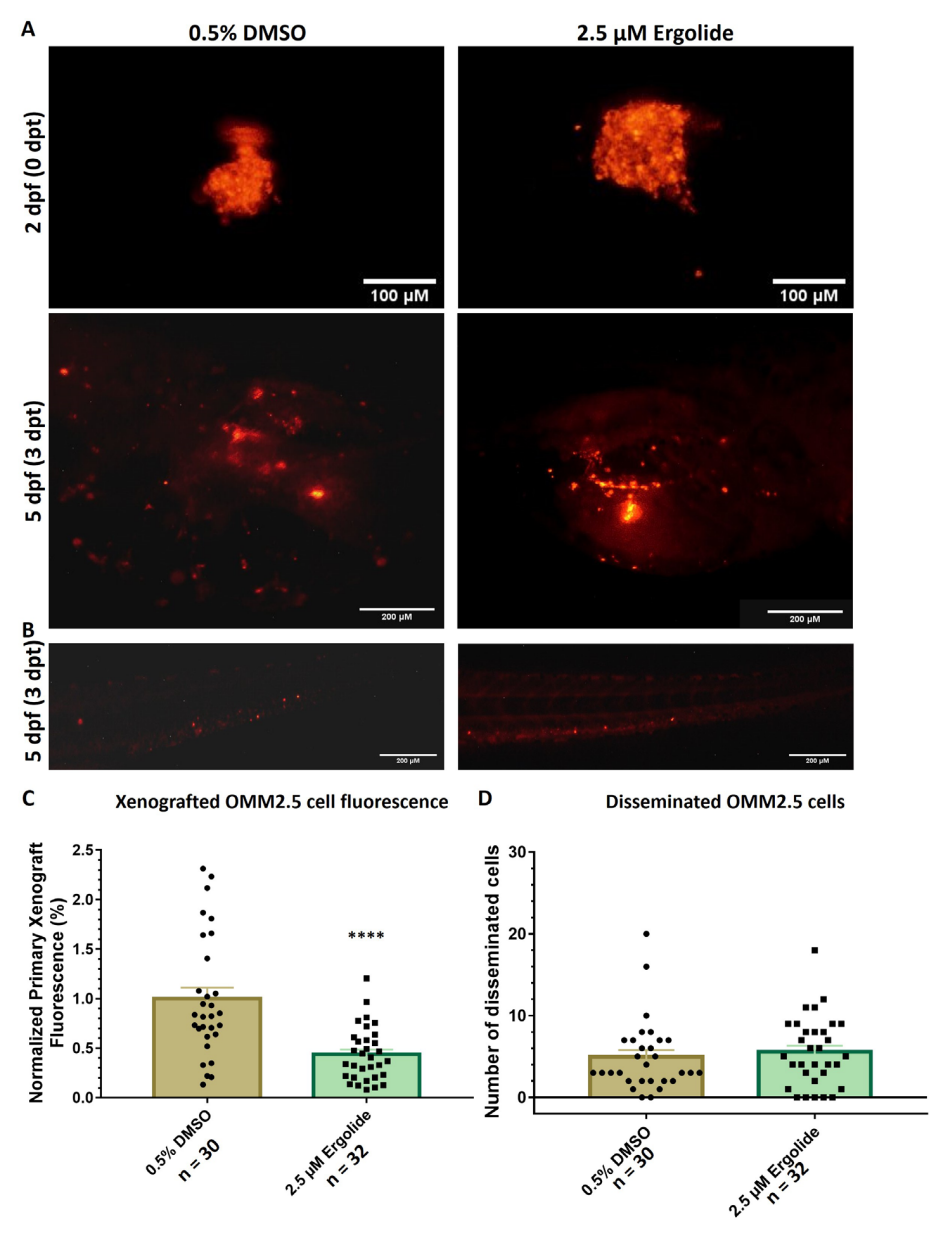
Anti-cancer effects of ergolide observed *in vivo* in zebrafish OMM2.5 xenograft models. ( **A**, **C**) Representative images of zebrafish larvae transplanted with OMM2.5 ^Dil^ labelled cells at 2 days post fertilization (dpf, top panel); representative images of zebrafish larvae transplanted with OMM2.5 ^Dil^ labelled cells at 5 dpf, 3 days post treatment (dpt, bottom panel). A 56% (****, *p*<0.0001%) reduction, on average, in primary xenograft fluorescence observed in the 2.5 μM ergolide (n = 32) treated samples compared to 0.5% DMSO (n = 30) treated samples. ( **B**, **D**) A significant difference was not detected in the average number of disseminated cells. Statistical analysis performed using Student's T-test.

Using zebrafish xenograft models generated with transplanted OMM2.5 cells, the anti-cancer effect of ergolide *in vivo*, was assessed as described previously ^26^. An initial maximum tolerated dose screen was performed, with all tested concentrations up to 5 μM ergolide was well tolerated in wildtype zebrafish larvae (Supplementary Figure 1). Increased larval death was observed at treatment concentrations of 7.5 and 10 μM ergolide. OMM2.5 cells labelled with Dil were injected into the perivitelline space of 2-day-old larvae, after which the larvae were treated with 0.5% DMSO or 2.5 μM ergolide (selected based on *in vitro* data) for 3 days ( Figure 2). Larvae treated with 2.5 μM ergolide exhibited an average of 56% ( *p*<0.0001) reduction of normalized primary xenograft fluorescence in comparison to vehicle control ( Figure 2A and 2C). Given the short-term treatment regime, a significant difference between the number of OMM2.5 cells disseminated in vehicle control (averaging 5 cells) and ergolide (averaging 5.6 cells) treatment groups was not detected ( Figure 2B and 2D). Overall, given the treatment regime, ergolide resulted in a significant anti-cancer effect *in vivo*, without affecting the number of disseminated cells.

### Analysis of ergolide-treated OMM2.5 cells secretome profile

Knowing that inflammation plays a role in UM disease pathogenesis ^1, 44^ and ergolide is reported to exert anti-inflammatory responses in tumor cells ^16^, we postulated that ergolide may modulate inflammatory pathways to exert its observed anti-cell survival effects in OMM2.5 cells. Therefore, we analyzed changes in select inflammatory markers in the secretome of 2.5 μM ergolide treated OMM2.5 cells, using a Multiplex ELISA inflammatory panel. OMM2.5 cells were treated with 0.5% DMSO or 2.5 μM ergolide and the respective conditioned media collected at 4 and 24 hours post treatment. Out of 54 analytes measured, 12 were detected in all samples measured. No significant changes were observed in any of the twelve analytes between 0.5% DMSO or 2.5 μM ergolide treated samples, after 4 or 24 hours post treatment, given the treatment regime undertaken (Supplementary Figure 2).

### Understanding Ergolide mechanism of action through proteome profiling of whole OMM2.5 cell lysates

**Figure 3.**
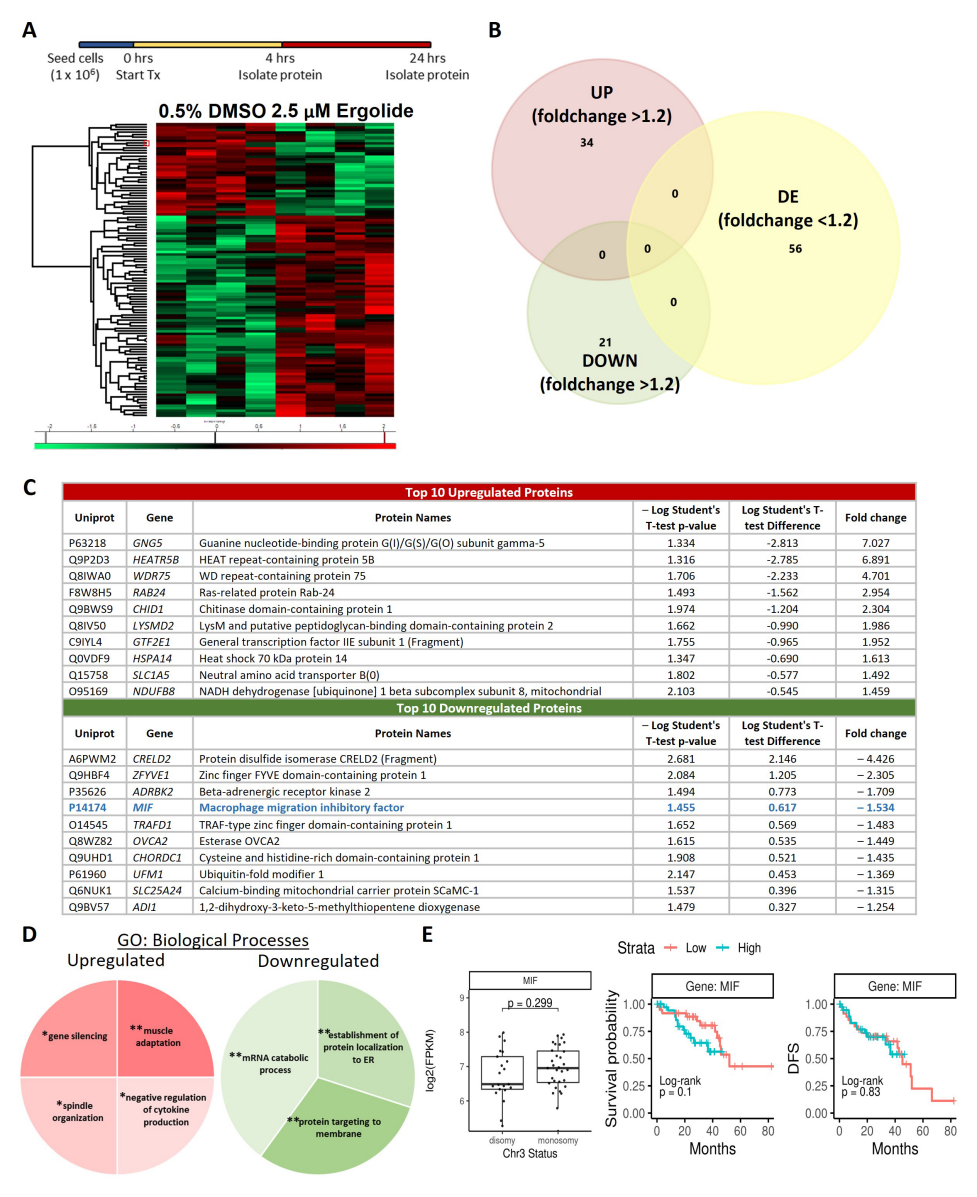
Proteome profiling following 24 hours ergolide treatment of OMM2.5 cells. ( **A**) Schematic diagram depicting treatment regime and heatmap highlighting differentially expressed proteins. ( **B**) Venn diagram showing the number of proteins significantly differentially expressed (up/down) or differentially expressed (DE), given a cutoff of *p*≤0.05 and a ≥ 1.2 fold change. ( **C**) Table of top ten proteins that are significantly up- or down-regulated following 24 hours of ergolide treatment. ( **D**) Pathways enriched for GO: Biological processes. ( **E**) TCGA analysis of UM patient samples comparing *MIF* expression levels to chromosome 3 (Chr 3) status, overall survival and disease-free survival (DFS), (n = 80).

As an alternative to understand the molecular changes induced by ergolide in OMM2.5 cells, proteome-profiling was performed on whole cell extracts at 4 and 24 hours post treatment. 5023 proteins were identified in total across the samples with 52 and 55 proteins, significantly altered at 4 hours and 24 hours, respectively, applying stringency cut-offs of *p* value < 0.05 and fold-change ≥+ 1.2 ( Figure 3, Supplementary Table 1 and Supplementary Figure 3). Comparing the datasets, two proteins, heme oxygenase 1 (HMOX1) and zinc finger FYVE domain-containing protein 1 (ZFYVE) were found to be significantly differentially expressed at both 4 and 24 hours of ergolide treatment (Supplementary Figure 3A). Treatment of OMM2.5 cells with 2.5 µM ergolide for 4 hours resulted in 29 proteins significantly upregulated and 23 proteins significantly downregulated (Supplementary Figure 3B). From the analysis we identified HMOX1 and growth/differentiation factor 15 (GDF15) as significantly upregulated, by ergolide (Supplementary Figure 3C). These proteins have been implicated in cancer and in UM pathogenesis ^45– 49^. Furthermore, validation of HMOX1 ( *p* = 0.047) and GDF15 ( *p* = 0.026) expression levels by immunoblotting corroborated our proteomics data findings (Supplementary Figure 3D). Cluego GO: biological processes pathway analysis indicated that *Golgi vesicle transport* (45.45%; upregulated), *retrograde vesicle-mediated transport/Golgi to endoplasmic reticulum* (27.27%; upregulated), *vesicle-mediated transport to the plasma membrane* (27.27%; upregulated), *organic acid catabolic process* (50%; downregulated) and *acyl-CoA metabolic* (50%; downregulated) processes were significantly altered (Supplementary Figure 3E).

Following 24 hours of 2.5 µM ergolide treatment, 34 and 21 proteins were significantly up- or down-regulated, respectively ( Figure 3A and Figure 3B). Guanine nucleotide-binding protein G(I)/G(S)/G(O) subunit gamma-5 (GNG5; 7.027-fold), HEAT repeat-containing protein 5B (HEATR5B; 6.891-fold), WD repeat-containing protein 75 (WDR75; 4.701-fold), Ras-related protein Rab-24 (RAB24; 2.954-fold), chitinase domain-containing protein 1 (CHID1; 2.304-fold), LysM and putative peptidoglycan-binding domain-containing protein 2 (LYSMD2; 1.986-fold), general transcription factor IIE subunit 1 (Fragment) (GTF2E1; 1.952-fold), heat shock 70 kDa protein 14 (HSPA14; 1.613-fold), neutral amino acid transporter B(0) (SLC1A5; 1.492-fold) and NADH dehydrogenase [ubiquinone] 1 beta subcomplex subunit 8, mitochondrial (NDUFB8; 1.459-fold) were in the top 10 most upregulated proteins ( Figure 3C). The top most downregulated proteins included protein disulfide isomerase CRELD2 (Fragment) (CRELD2; 4.426-fold), zinc finger FYVE domain-containing protein 1 (ZFYVE; 2.305-fold), beta-adrenergic receptor kinase 2 (ADRBK2; 1.709-fold), macrophage migration inhibitory factor (MIF; 1.534-fold), TRAF-type zinc finger domain-containing protein 1 (TRAFD1; 1.483-fold), esterase OVCA2 (OVCA2; 1.449-fold), cysteine and histidine-rich domain-containing protein 1 (CHORDC1; 1.435-fold), ubiquitin-fold modifier 1 (UFM1; 1.369-fold), calcium-binding mitochondrial carrier protein SCaMC-1 (SLC25A24; 1.315-fold) and 1,2-dihydroxy-3-keto-5-methylthiopentene dioxygenase (ADI1; 1.254-fold) ( Figure 3C). Pathway analysis showed that processes such as *gene silencing* (25%), *muscle adaptation* (25%), *spindle organization* (25%) and *negative regulation of cytokine production* (25%) were upregulated ( Figure 3D). While proteins involved in *mRNA catabolic process* (40%), *establishment of protein localization to endoplasmic reticulum* (30%) and *protein targeting to membrane* (30%) were downregulated ( Figure 3D). Given that MIF has been implicated in UM/MUM pathogenesis ^50– 53^, *MIF* expression level was analyzed for correlation to chromosome 3 status, overall survival and disease-free survival in the TCGA-UM dataset (n = 80). A significant difference between high or low *MIF* expression levels correlating to overall survival (OS) or disease-free survival (DFS) was not detected ( Figure 3E). Additionally, there was no difference between chromosome 3 status of the UM samples and *MIF* expression levels ( Figure 3E).

### Isolation of extracellular vesicles from OMM2.5 cells treated with Ergolide

**Figure 4.**
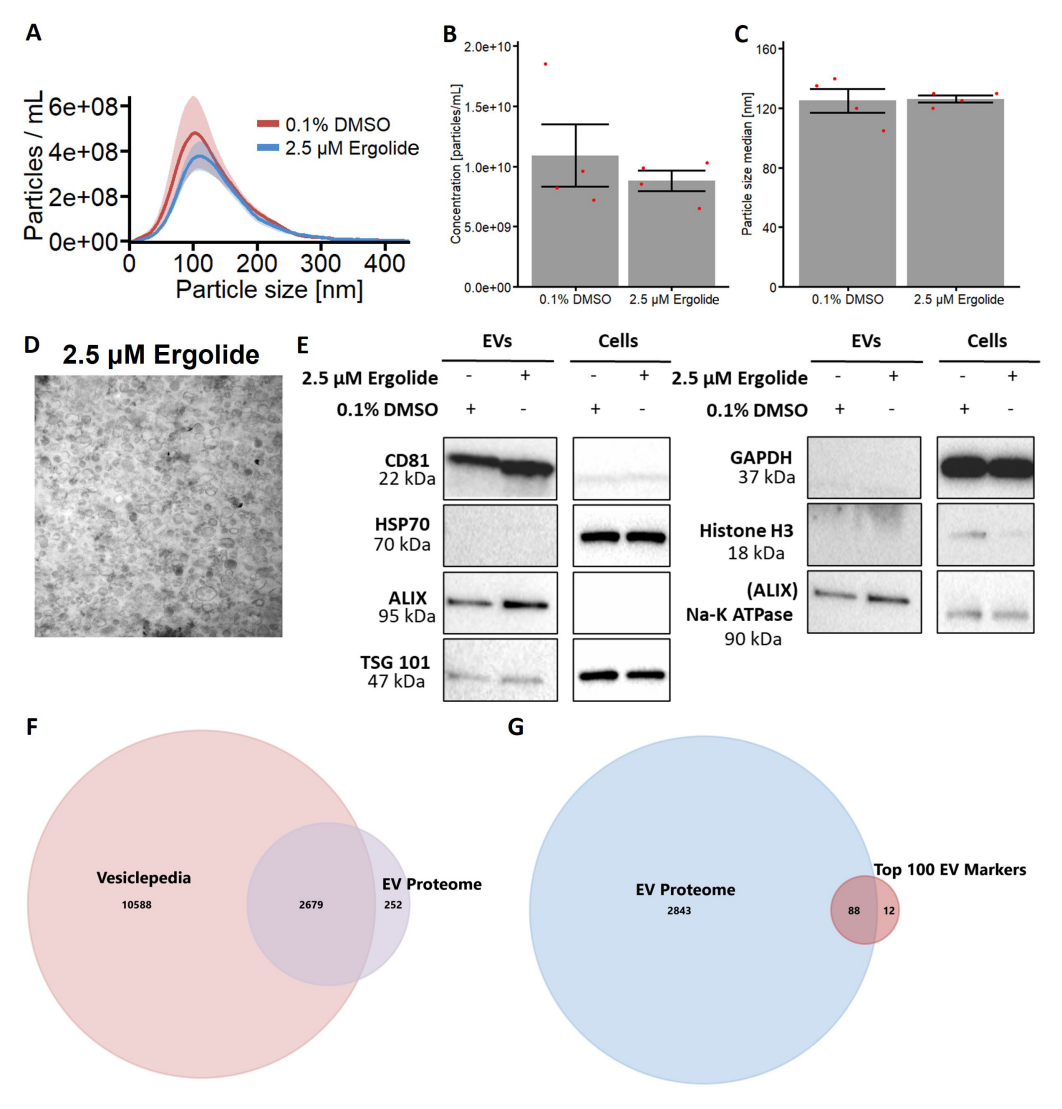
Characterization of EV isolated from OMM2.5 cells. ( **A**) NTA analysis of isolated OMM2.5 EV particle size ranging from 50 nm to 250 nm in both 0.1% treated DMSO and 2.5 μM ergolide treated samples. ( **B** and **C**) A significant difference in the amount of particles and particle size was not observed between ergolide treated samples and vehicle control. ( **D**) TEM image of particles isolated from OMM2.5 cells. ( **E**) Immunoblot analysis of select proteins in OMM2.5 EV isolates and cell lysate (N = 1). ( **F**) Comparison between Vesiclepedia compendium and EV proteome. ( **G**) 88 proteins from the list of top 100 EV markers identified in OMM2.5 EV proteome.

Increasing evidence indicates extracellular vesicles (EVs) influence the regulation of cancer progression ^20, 54^. We investigated OMM2.5 EVs to further understand the proteome of EVs, to address whether the EV profile is modulated by ergolide treatment and to identify novel factors and therapeutic targets involved in MUM disease pathogenesis. Hence, we isolated EVs from OMM2.5 cells to *i)* evaluate the proteome of these UM-related EVs and *ii)* determine whether ergolide treatment altered the OMM2.5 EV proteome. Briefly, OMM2.5 cells were seeded overnight and treated with 0.1% DMSO or 2.5 μM ergolide in serum-free media for 24 hours. EVs were isolated from the cell culture supernatant with differential centrifugation and characterized according to the Minimal information for studies of extracellular vesicles 2018 (MISEV2018) guidelines ^55^. The EV isolates contained particles with a diameter of mostly 50 - 250 nM which is in the expected size range for small EVs ( Figure 4A). Ergolide treatment did not affect the concentration or size distribution of the isolated particles ( Figure 4B and Figure 4C). Membrane vesicles were identified in the isolated fractions by transmission electron microscopy ( Figure 4D). Additional characterization by immunoblotting revealed EV isolates were positive for EV markers ( *e.g.* CD81, Alix) and negative for non-vesicular markers ( *e.g.* histone-H3) ( Figure 4E). A total of 2931 proteins (mapped by gene names) were identified from the EV isolates, of which 2679 are established proteins in Vesiclepedia and 252 are potentially novel EV proteins ( Figure 4F). When compared with the top 100 EV markers listed in Vesiclepedia, 88 common proteins were identified in the EVs isolated from OMM2.5 cells indicating a clear enrichment in EV proteins and validating our approach to study the effect of ergolide in EVs isolated from UM cell lines ( Figure 4G and Supplementary Table 2).

### Proteome profiling of extracellular vesicles following treatment with Ergolide

**Figure 5.**
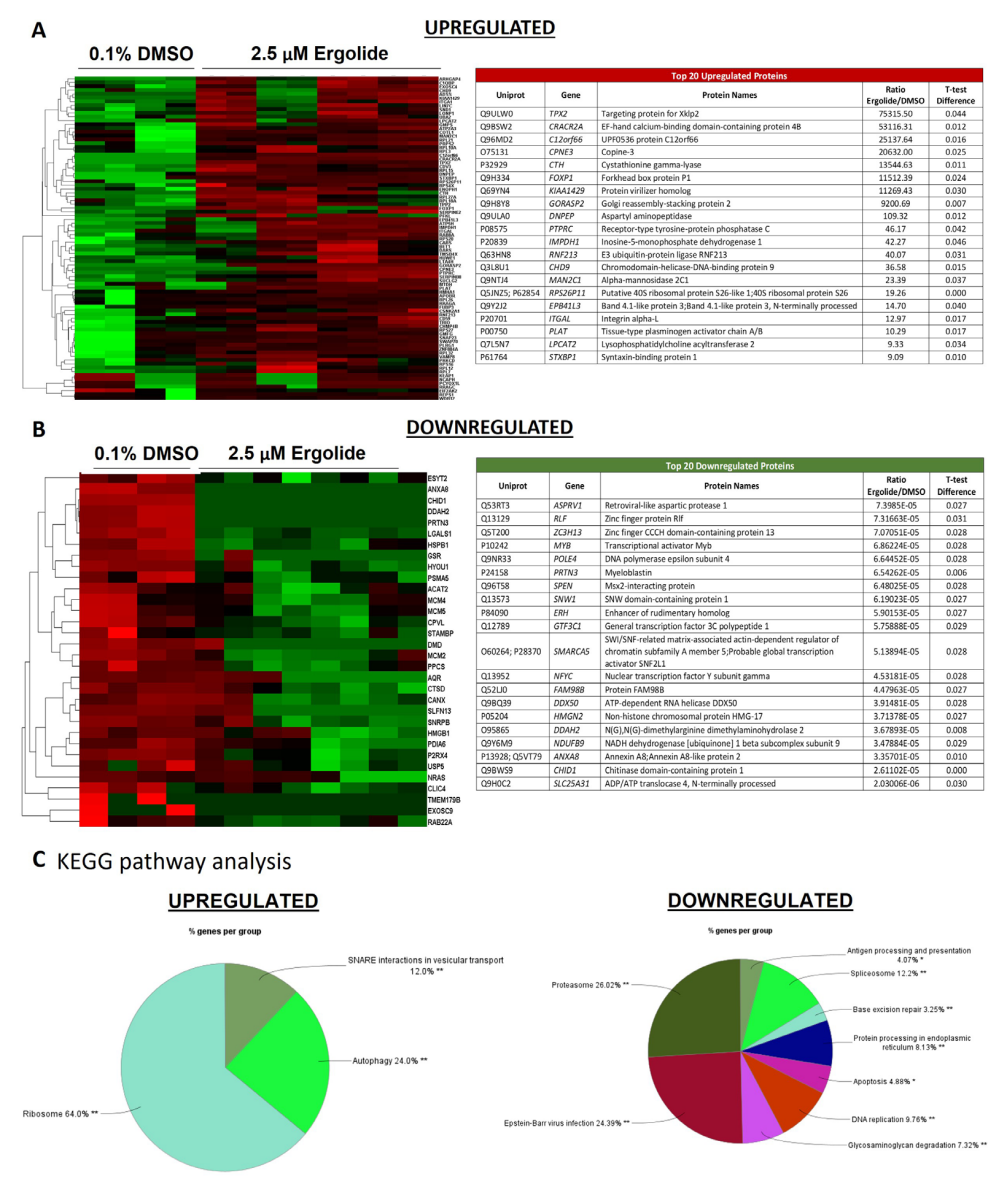
Analysis of differentially expressed proteins in EVs isolated from OMM2.5 cells treated with ergolide. ( **A**) Heatmap of upregulated proteins in ergolide treatment group (left panel); list of top 20 significantly upregulated proteins in samples treated with ergolide (right panel). ( **B**) Heatmap of downregulated proteins in ergolide treatment group (left panel); list of top 20 significantly downregulated proteins in samples treated with ergolide (right panel). ( **C**) KEGG pathway analysis to determine pathways enriched in EVs following ergolide treatment.

The EV proteome was analyzed to uncover alterations caused by ergolide treatment. Compared to the vehicle control EVs, ergolide treated EVs displayed 315 proteins differentially expressed by using the statistical threshold α = 0.05 and minimal fold-change of > + 1.5 ( Figure 5). Of the significantly altered proteins, 83 were upregulated and 232 proteins were down-regulated. The top 10 up- and down-regulated proteins were determined to be, targeting protein for Xklp2 (TPX2), EF-hand calcium-binding domain-containing protein 4B (CRACR2A), UPF0536 protein C12orf66 (C12orf66), Copine-3 (CPNE3), Cystathionine gamma-lyase (CTH), Forkhead box protein P1 (FOXP1), Protein virilizer homolog (KIAA1429), Golgi reassembly-stacking protein 2 (GORASP2), Aspartyl aminopeptidase (DNPEP) and Receptor-type tyrosine-protein phosphatase C (PTPRC) as upregulated; ADP/ATP translocase 4, N-terminally processed (SLC25A31), Chitinase domain-containing protein 1 (CHID1), Annexin A8;Annexin A8-like protein 2 (ANXA8), NADH dehydrogenase [ubiquinone] 1 beta subcomplex subunit 9 (NDUFB9), N(G),N(G)-dimethylarginine dimethylaminohydrolase 2 (DDAH2), Non-histone chromosomal protein HMG-17 (HMGN2), ATP-dependent RNA helicase DDX50 (DDX50), Protein FAM98B (FAM98B), Nuclear transcription factor Y subunit gamma (NFYC) and SWI/SNF-related matrix-associated actin-dependent regulator of chromatin subfamily A member 5;Probable global transcription activator SNF2L1 (SMARCA5) as downregulated ( Figure 5A and 5B).

KEGG pathway analysis of enriched proteins highlighted proteins involved in Ribosome, Autophagy and SNARE interactions in vesicular transport to be upregulated and proteins involved in processes such as proteasome, spliceosome, antigen processing and presentation, DNA replication, apoptosis, base excision repair, protein processing in endoplasmic reticulum, and glycosaminoglycan degradation, to be downregulated ( Figure 5C, Figure 5D, Supplementary Table 3 and Supplementary Table 4). Within the highly enriched proteins, several of them were also identified to be associated with vesicles (Supplementary Figure 4A and Supplementary Figure 5A). Additional Reactome pathway analysis included terms such as cellular response to starvation, mTORC1 mediated signaling and formation of ternary complex and subsequently 43S complex to be highly enriched in ergolide treated samples (Supplementary Figure 4B). Within the downregulated proteins, CORUM analysis indicated terms such as 26S proteasome, MCM complex, splicing, DNA methylation, cell cycle arrest, cell cycle checkpoints large Dorsha complex and PA700 complex to be significantly downregulated in ergolide treated group (Supplementary Figure 5B). Taken together, ergolide treatment resulted in a substantial modulation of cellular processes related to proteasome function, senescence and cell death/survival, and these changes were detectable by the changes in EV proteome as well.

**Figure 6.**
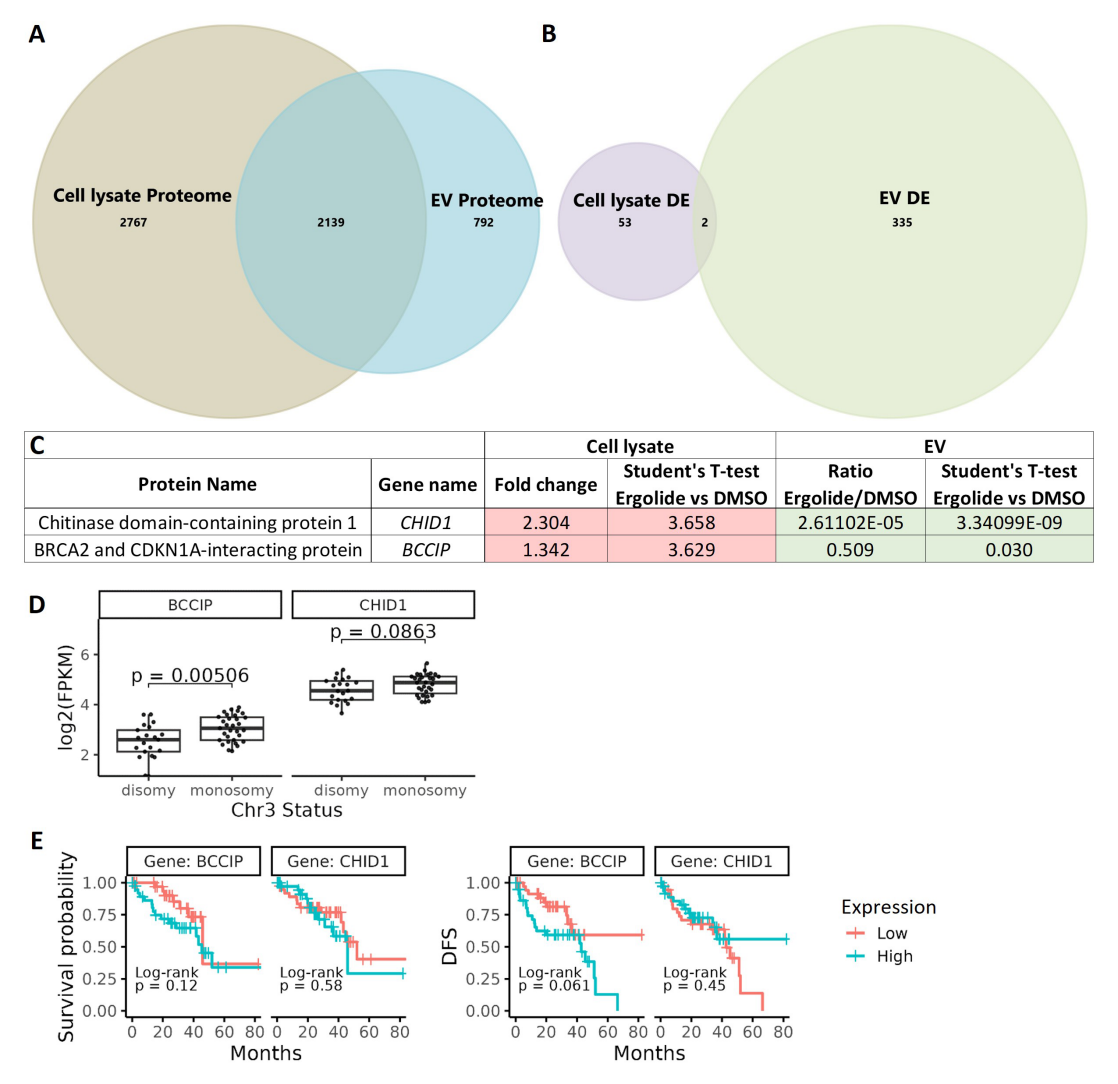
Comparison of cellular lysate and EV proteome. ( **A**) Venn diagram depicting 2139 common proteins in the proteome of OMM2.5 cell lysate and OMM2.5 EV samples. ( **B**) Among the differentially expressed proteins, two common proteins were identified in the OMM2.5 cell lysate and EV samples. ( **C**) Table presenting CHID1 and BCCIP as significantly upregulated (red) in ergolide treated OMM2.5 cell lysate and downregulated (green) in EVs isolated from ergolide treated OMM2.5 cells. ( **D**) Higher *BCCIP* ( *p* = 0.005) expression levels noted in chromosome 3 (Chr 3) monosomy UM patients, with no difference in *CHID1* expression level and Chr 3 status. ( **E**) High or low expression levels of *BCCIP* and *CHID1* did not correlate to overall survival or disease-free survival (DFS) in UM TCGA patient samples.

A comparative analysis between cell lysate proteome and EV proteome identified 2139 common proteins ( Figure 6A). From the differentially expressed proteins, BCCIP (BRCA2 and CDKN1A interacting protein) and CHID1 (chitinase domain containing 1) were the only ones differentially expressed by ergolide in both the cellular and (extracellular) EV isolates ( Figure 6B). Interestingly, CHID1 and BCCIP were upregulated in the ergolide-treated cell lysate samples and in contrast, both proteins were significantly downregulated in the ergolide-treated EV isolates ( Figure 6C). Since specific reciprocal enrichment of these two proteins were observed between the two proteome datasets, the expression level of the mRNA transcripts encoding these proteins was analyzed in the TCGA-UM dataset (n = 80). Significant differences between *CHID1* expression levels to chromosome 3 status ( *p*=0.08), OS ( *p*=0.58) or DFS ( *p* = 0.45) were not observed ( Figure 6D and Figure 6E). However, significantly higher transcript levels of *BCCIP* ( *p* = 0.005) was found in UM patients with chromosome 3 monosomy compared to chromosome 3 disomy ( Figure 6D), and this monosomy is associated with worse UM prognosis and high risk of UM metastasis ^56– 58^. Differences between primary UM patients with low or high transcript levels of *BCCIP* did not reach significance in relation to OS or DFS ( Figure 6E). However, there was a strong tendency of patients with higher *BCCIP* ( *p* = 0.061) expression levels to have lower DFS ( Figure 6E). 

## Discussion

Metastatic UM is a poor prognosis cancer with limited treatment options currently available. In our exploratory study, we discovered that the plant-based compound, ergolide, has potential therapeutic effects in UM/MUM cells. We show ergolide elicits significant reductions in UM and MUM cell survival *in vitro*; and a marked reduction in primary OMM2.5 xenograft fluorescence in zebrafish.

A key knowledge gap is to better understand the molecular mechanism of action of ergolide. Some evidence links ergolide with the NF-κB signaling pathway ^16, 18, 19^. Significantly, there is evidence that the NF-κB pathway is disease relevant in UM. The canonical and non-canonical NF-κB signaling pathway(s) are implicated in the regulation of UM cell proliferation and apoptosis ^59– 61^. The transcription factors, NF-κB1 (p50/p105), NF-κB2 (p52/p100) and related proteins, transcription factor p65 (RELA), transcription factor RELB (RELB), and NF-κB inducing kinase (NIK) are expressed in primary UM and liver metastatic tumors, with higher levels of NF-κB2, RELB, and NIK reported in UM liver metastatic samples compared to UM primary tumors ^59, 62^. Recent findings by Souri *et al.*, concluded that loss of chromosome 3 and BAP1 expression correlated with the upregulation of the NF-κB (NF-κB1, NF-κB2, and RELB) signaling pathway and consequently promotes inflammation in UM tumors ^63^. In cancer, the NF-κB signaling pathway plays a multifaceted role in cell proliferation, differentiation, cell survival, apoptosis, angiogenesis and inflammatory response ^64– 66^. NF-κB may even exert tumor suppressive activities in certain cancer ^67^. Here, we assessed a panel of inflammatory markers including TNF-α, IL-1, IFN-γ, IL-6 and VEGF, by ELISA to determine whether ergolide modulated the OMM2.5 inflammatory secretome. However, no significant change in any of the tested panel of inflammatory markers was observed. The involvement of the NF-κB signaling pathway and/or other inflammatory pathways cannot be ruled and need to be explored further. It is also true that ergolide’s mechanism of action is not fully elucidated and other targets and pathways may play key effector roles.

Consequently, in order to investigate ergolide’s mechanism of action using an unbiased omics approach, the whole soluble cell proteome of OMM2.5 cells treated with ergolide was profiled after 4 and 24 hours. Potential mechanisms of action were uncovered. Pathways such as vesicle-mediated transport, gene silencing and negative regulation of cytokine production were significantly altered by ergolide. Interestingly, macrophage migration inhibitory factor (MIF) was significantly downregulated following 24 hours of ergolide treatment. MIF, a proinflammatory cytokine, is well known for its essential roles in inflammatory and immune responses ^68– 70^. MIF also plays a multifaceted role promoting tumor progression, metastasis and angiogenesis, in the context of cancer ^45, 71, 72^. Several studies show that high MIF expression levels contribute to aggressive disease and poor prognosis cancer(s) ^45, 73– 75^. Previously, it was reported that UM cells produced MIF as a protective mechanism against cell lysis by natural killer cells ^50^. The authors presented that cell lines derived from UM metastases produced almost double the amount of active MIF compared to primary UM cell lines. Another study demonstrated that M2 macrophages present within MUM liver tumors, have elevated *MIF* expression levels ^51^. MIF was also found to be upregulated in the aqueous humor of UM patients ^52^. Furthermore, analysis of the TCGA dataset indicated that approximately 9% of the samples had high *MIF* expression levels. From our TCGA UM-dataset analysis, we did not observe a significant difference between *MIF* expression levels to OS, DFS or chromosome 3 status. Ambrosini *et al.*, showed that MIF is overexpressed in UM cells and in exosomes derived from UM cells ^53^. They also demonstrated that treatment of UM cells with MIF inhibitors or MIF depleted exosomes, blocked cell migration and invasion stimulated by exosome treated hepatocytes. Furthermore, a significant reduction in metastasis was observed in a metastatic mice model co-treated with UM exosomes and a MIF inhibitor compared to mice only treated with UM exosomes, highlighting the vital role of MIF in UM tumor metastasis ^53^. In our study, MIF was also present in the EV proteome, but unlike in the cell lysate, EV MIF was not significantly altered upon ergolide treatment. In summary, ergolide significantly reduces cellular MIF levels, and MIF is linked to UM cell metastasis.

EVs are modulated in cancers and perform crucial roles in tumorigenesis processes; with increased number of circulating EVs reported in patients ^20, 54, 76, 77^. EVs also have potential as vehicles for cancer drug delivery ^78^. We hypothesized that in addition to modulating the cell proteome, ergolide may also modulate the EV proteome. Here, we successfully isolated and characterized small EVs from OMM2.5 cells. Tsering *et al.*, reported EVs of a mean diameter of 181 nm to 214 nm, and ~4 × 10 ^9^ particles isolated in various UM/MUM cells ^79^. We corroborate these findings in our study with similar particle concentrations, albeit slightly smaller size EVs, isolated. Analysis of the OMM2.5 cell, EV proteome identified many identical EV proteins as reported by Tsering *et al*., validating our methodology. For example, nidogen1 (NID1), a potential biomarker for metastatic breast cancer and melanoma, was highly expressed in their EVs from OMM2.5 cells and was also present in our proteomics dataset ^79, 80^. Moreover, in our data, altered pathways included processes such as ribosome, autophagy, SNARE complex, proteasome, spliceosome, antigen processing and presentation, DNA replication, apoptosis and glycosaminoglycan degradation. Changes to these proteins/processes are not surprising as in cancer, there is increased production of proteins/ribosomal proteins and consequently cells have to eliminate these byproducts ^81, 82^.

Intriguingly, under our ergolide treatment regime, BCCIP and CHID1 protein expression levels were significantly upregulated in ergolide treated OMM2.5 cellular lysates, but remarkably, the two proteins were inversely, significantly downregulated in EVs lysates isolated from ergolide treated OMM2.5 cells. The role of BCCIP and CHID1 in EV biology and pathology is unclear. However, BCCIP was previously identified in EVs from A431 squamous carcinoma cells ^83^. Likewise, *CHID1* was previously identified in the exosomal transcriptome of a hepatocellular carcinoma (HKCI-8) cell line ^84^. Collectively, these support the need for further investigation of BCCIP and CHID1 in EVs. However, in relation to cancer pathology and prognostication, there is precedence for the relevance of BCCIP or CHID1. Several studies report BCCIP mediates important roles in mitosis, cell cycle regulation, chromosome stability, homologous recombination-dependent DNA repair, suppression of DNA replication stress, and ribosome biogenesis ^85– 87^. Dysregulation of BCCIP is associated with hepatocellular carcinoma, colorectal cancer tissue and renal cell carcinoma tissue ^88, 89^. Elevated BCCIP expression levels are correlated to a prognosis of poorer overall patient survival in lung adenocarcinoma and esophageal squamous cell carcinoma ^90, 91^. CHID1 is reported to function as a regulator of the inflammatory response elicited by macrophages ^92^. High CHID1 levels are associated with low survival rate and poor survival prognosis in colorectal cancer patients ^93^. In non-small cell lung cancer and adenocarcinoma, high CHID1 levels are indicative of good prognosis and suitable as a prognostic marker ^94^. To date, there is limited understanding of the relevance of BCCIP or CHID1 in UM. Hence, we investigated the possibility of BCCIP and CHID1 as potential prognostic biomarkers in UM/MUM. However, from the TCGA transcriptomic data, no significant correlations occurred between *BCCIP* or *CHID1* transcript levels, and overall survival or disease-free survival in 80 UM patients. However, the trend between high *BCCIP* transcript levels and increased metastatic UM is worthy of further analysis in other UM cohorts and by distinct correlations to protein levels of BCCIP. Notably, higher *BCCIP* expression levels were associated with chromosome 3 monosomy, which is the aggressive form of UM with poor survival prognosis ^56– 58, 95^.

Ergolide shows promise as a therapeutic option for UM/MUM that needs to be thoroughly investigated. Understanding the complex tumor microenvironment may indeed be the key to uncovering novel therapeutic targets, biomarkers and future treatment options for this rare disease.

## Data Availability

Zenodo: Ergolide Mediates Anti-Cancer Effects on Metastatic Uveal Melanoma Cells and Modulates their Cellular and Extracellular Vesicle proteomes, https://doi.org/10.5281/zenodo.7844457 ^96^. This project contains the following underlying data: Figure 1A_1B_MTT assay.pzfx Figure 1D_Clonogenic Assay.pzf Figure 2C_2D_Xenografts Assay.pzf Figure 4E_Immunoblot.jpg Extended Figure 2_Secretome ELISA.pzfx Zenodo: Ergolide Mediates Anti-Cancer Effects on Metastatic Uveal Melanoma Cells and Modulates their Cellular and Extracellular Vesicle proteomes, https://doi.org/10.5281/zenodo.7844457 ^96^. This project contains the following extended data: Extended Dataset Figures.pdf (Supplementary information to “Ergolide Mediates Anti-Cancer Effects on Metastatic Uveal Melanoma Cells and Modulates their Cellular and Extracellular Vesicle proteomes” article. Inclusive of figures, table and legends.) Extended Data Table 1 - OMM2.5 cell lysate.xlsx Extended Data Table 2, 3, 4 - OMM2.5 EV.xlsx Data are available under the terms of the Creative Commons Attribution 4.0 International license (CC-BY 4.0).
