# Supplementary material for: Ergolide mediates anti-cancer effects on metastatic uveal melanoma cells and modulates their cellular and extracellular vesicle proteomes
Source: Open Res Eur. 2023 Nov 13;3:88. Originally published 2023 Jun 8. [Version 2] doi: 10.12688/openreseurope.15973.2 (PMC10654492; doi:10.12688/openreseurope.15973.2)
Supplement: Peer review history

# Reviewer report: Reviewer response for version 2

**DOI:** 10.21956/openreseurope.18171.r36207

**Published:** 2023-11-16

**Authors:** Shivani Malpotra

**Affiliations:** 1 Ophthalmology, Mayo Clinic Minnesota, Rochester, Minnesota, USA

Author has incorporated all the suggestions and changes that were raised at the time of review.

Is the study design appropriate and does the work have academic merit?

Yes

Is the work clearly and accurately presented and does it cite the current literature?

Yes

If applicable, is the statistical analysis and its interpretation appropriate?

I cannot comment. A qualified statistician is required.

Are all the source data underlying the results available to ensure full reproducibility?

Yes

Are the conclusions drawn adequately supported by the results?

Yes

Are sufficient details of methods and analysis provided to allow replication by others?

Yes

Reviewer Expertise:

Ocular oncology- Retinoblastoma, Uveal melanoma & extracellular vesciles.

I confirm that I have read this submission and believe that I have an appropriate level of expertise to confirm that it is of an acceptable scientific standard.

# Reviewer report: Reviewer response for version 1

**DOI:** 10.21956/openreseurope.17247.r35099

**Published:** 2023-10-03

**Authors:** Shivani Malpotra

**Affiliations:** 1 Ophthalmology, Mayo Clinic Minnesota, Rochester, Minnesota, USA

The manuscript is well-written with a focus on using anti-cancer properties of plant-based compound Ergolide on uveal melanoma cell lines (in vitro) and zebrafish metastatic uveal melanoma xenograft (in vivo).  The author has also explored the effect of this compound on proteome of cellular and extracellular vesicles. All the sections of this manuscript have been elaborated properly with sufficient details in materials & methods. Although the manuscript shows very promising results, there are some minor concerns that needs to be addressed before this manuscript is ready to be indexed. Please find some minor revisions comments as follows:

1. Although the author mentioned in few places that proteomic profiling was done to understand the mechanism of action of Ergolide, but it still not quite clear in the manuscript. A little conclusive diagram of mechanism of action with all the proteins involved would really enhance the quality of the work.
2. Suggestion: If it allows the authors to carry out some gene expression analysis from the same set of samples (control vs. treatment), protein profiling can be correlated with it to draw some better conclusions.

Is the study design appropriate and does the work have academic merit?

Yes

Is the work clearly and accurately presented and does it cite the current literature?

Yes

If applicable, is the statistical analysis and its interpretation appropriate?

I cannot comment. A qualified statistician is required.

Are all the source data underlying the results available to ensure full reproducibility?

Yes

Are the conclusions drawn adequately supported by the results?

Yes

Are sufficient details of methods and analysis provided to allow replication by others?

Yes

Reviewer Expertise:

Ocular oncology- Retinoblastoma, Uveal melanoma & extracellular vesciles.

I confirm that I have read this submission and believe that I have an appropriate level of expertise to confirm that it is of an acceptable scientific standard, however I have significant reservations, as outlined above.

# Reviewer report: Reviewer response for version 1

**DOI:** 10.21956/openreseurope.17247.r32619

**Published:** 2023-08-01

**Authors:** Alexandre P. Moulin

**Affiliations:** 1 Jules-Gonin Eye Hospital, Fondation Asile des Aveugles, University of Lausanne, Lausanne, Switzerland

In this manuscript, the authors assessed the effects of ergolide on primary and metastatic melanoma cell lines in vitro (Mel 285, Mel270 and OMM2.5) and in metastatic cell in vivo (OMM2.5) in zebrafish xenograft models. Using MMT and clonogenic assays, the authors demonstrated that ergolide reduced proliferation in the cell lines. In zebrafish xenografts, treatment with 2.5 µM ergolide for 3 days was associated with reduced fluorescence, but not with decreased number of xenograft tumor cells. Analysis of inflammatory secretome from OMM2.5 treated with or without ergolide did not reveal significant differences. The authors further assessed the changes in proteome in OMM2.5 induced by ergolide (4 and 24 hours) with upregulation of 34 and downregulation of 21 proteins (notably MIF) after 24 hours. They further isolated and characterized EV from OMM2.5 treated with ergolide. The authors identified 83 upregulated and 232 downregulated proteins in EV treated with ergolide with upregulation of ribosome, autophagy, mTORC1 signaling and downregulation of notably cycle cell arrest and DNA methylation. Comparing proteins identified in EV and cell lysates, the authors identified 2139 commonly expressed proteins and only 2 differentially expressed proteins: CHID1 and BCCIP. None of these genes had a significant impact on survival in the TCGA UM cohort.

 This manuscript is interesting, well written and the experiences are well conducted.

 A few comments:

- Did the authors assessed IC50 of ergolide for Mel270 and 285? Please specify why a treatment of 96 hours has been selected?
- It seems that the authors have not included a cell line with BAP1 mutation (e.g. MP46), seen in the majority of metastatic uveal melanoma. It would also be interesting to know the expression of BAP1 in Mel270 and OMM2.5, both derived from the same patient, as there was a mismatch between the primary tumor (BAP1 negative) and the primary (Mel270) and the metastasis (both BAP1 positive). Same applies to Mel285. The use of short-term UM cultures would have considerably strengthened the study.
- As the authors rightly pointed out, ergolide treatment has been described to activate NF- κβ pathway. Although the authors did not identify significant differences in inflammatory secretome of treated and untreated UM cell lines, it would be interesting to assess if NF- κβpathway is activated in tumor cells with ergolide treatment.

Is the study design appropriate and does the work have academic merit?

Yes

Is the work clearly and accurately presented and does it cite the current literature?

Yes

If applicable, is the statistical analysis and its interpretation appropriate?

Yes

Are all the source data underlying the results available to ensure full reproducibility?

Yes

Are the conclusions drawn adequately supported by the results?

Yes

Are sufficient details of methods and analysis provided to allow replication by others?

Yes

Reviewer Expertise:

ocular pigmented tumors

I confirm that I have read this submission and believe that I have an appropriate level of expertise to confirm that it is of an acceptable scientific standard.
